# Upregulation of mesencephalic astrocyte-derived neurotrophic factor in glial cells is associated with ischemia-induced glial activation

**DOI:** 10.1186/1742-2094-9-254

**Published:** 2012-11-23

**Authors:** Yujun Shen, Aimin Sun, Yunhong Wang, Daqin Cha, Haiping Wang, Facai Wang, Lijie Feng, Shengyun Fang, Yuxian Shen

**Affiliations:** 1 Biopharmaceutical Research Institute, Hefei 230032, P R China; 2 School of Basic Medical Sciences, Anhui Medical University, 81 Meishan Road, Hefei 230032, P R China; 3 Center for Biomedical Engineering and Technology, Baltimore, MD, 21201, USA; 4 Department of Physiology, University of Maryland School of Medicine, 725 W Lombard St, Baltimore, MD, 21201, USA

**Keywords:** Mesencephalic astrocyte-derived neurotrophic factor, Endoplasmic reticulum stress, Glial activation, Cerebral ischemia

## Abstract

**Background:**

Mesencephalic astrocyte-derived neurotrophic factor (MANF), a 20 kDa secreted protein, was originally derived from a rat mesencephalic type-1 astrocyte cell line. MANF belongs to a novel evolutionally conserved family of neurotrophic factors along with conserved dopamine neurotrophic factor. In recent years, ever-increasing evidence has shown that both of them play a remarkable protective role against various injuries to neurons *in vivo* or *in vitro*. However, the characteristics of MANF expression in the different types of glial cells, especially in astrocytes, remain unclear.

**Methods:**

The model of focal cerebral ischemia was induced by rat middle cerebral artery occlusion. Double-labeled immunofluorescent staining was used to identify the types of neural cells expressing MANF. Primarily cultured glial cells were used to detect the response of glial cells to endoplasmic reticulum stress stimulation. Propidium iodide staining was used to determine dead cells. Reverse transcription PCR and western blotting were used to detect the levels of mRNA and proteins.

**Results:**

We found that MANF was predominantly expressed in neurons in both normal and ischemic cortex. Despite its name, MANF was poorly expressed in glial cells, including astrocytes, in normal brain tissue. However, the expression of MANF was upregulated in the glial cells under focal cerebral ischemia, including the astrocytes. This expression was also induced by several endoplasmic reticulum stress inducers and nutrient deprivation in cultured primary glial cells. The most interesting phenomenon observed in this study was the pattern of MANF expression in the microglia. The expression of MANF was closely associated with the morphology and state of microglia, accompanied by the upregulation of BIP/Grp78.

**Conclusions:**

These results indicate that MANF expression was upregulated in the activated glial cells, which may contribute to the mechanism of ischemia-induced neural injury.

## Background

Mesencephalic astrocyte-derived neurotrophic factor (MANF), a 20 kDa secreted protein, was initially isolated and purified from culture medium of immortalized rat type-1 astrocytes (also named ventral mesencephalic cell line 1 (VMCL1)) [1]. The gene encoding MANF is located in human chromosomal band 3p21 [2], and highly conserved gene mutations were detected in early-stage tumors [3]. The MANF gene was therefore also named arginine-rich, mutated in early stage of tumors (ARMET or ARP). However, the variations were found to be normal polymorphisms rather than tumor-specific mutations shortly after the report [4]. Subsequent studies showed that MANF protein does not contain an arginine-rich region and has neurotrophic effects with selectivity for dopaminergic neurons [1,5]. MANF, together with conserved dopamine neurotrophic factor (CDNF), belongs to a novel and evolutionally conserved family of neurotrophic factors [5]. Human MANF shares 59% amino acid identity with CDNF. The study on MANF solution structure revealed that MANF is composed of two domains, a saposin-like N-terminal domain and a well-conserved flexible C-terminal domain with a cysteine bridge [6,7]. The cysteine bridge may be involved in catalyzing the formation of intramolecular disulfide bonds and protein folding in the endoplasmic reticulum (ER) because of its similarity to the active site motif of thiol/disulfide oxidoreductases [8,9].

MANF is an ER stress inducible protein [5,10]. MANF expression was upregulated by various ER stress inducers in neural cell lines and in the cerebral ischemic tissue *in vivo*[5,10]. Similarly, MANF mRNA increased after brain ischemia and epileptic insults in the hippocampus and in the cerebral cortex [11]. Increasing evidence indicates that MANF plays a remarkable protective role against various injuries to neurons *in vivo* or *in vitro*[1,10,11]. Recombinant MANF promoted neuron proliferation and prevented neuron apoptosis induced by tunicamycin [12]. The upregulation of MANF after insults could therefore possibly result from activation of endogenous neuroprotective processes.

Glial cells, especially astrocytes, are the major component of neural tissues. These cells are critical participants in every major aspect of brain development, function, and disease. MANF is so named because of its origin [1]. As mentioned above, the induction and neuroprotection of MANF in neurons have been well demonstrated. However, the characteristics and the elaborate patterns of MANF expression in glial cells, especially in the astrocytes, have not been reported until now. In this study, the profiles of MANF expression and induction *in vivo* and *in vitro* were investigated in three types of glial cells, including astrocytes, microglia, and oligodendrocytes. In addition, the accompanied ER stress-induced glial death was also examined.

## Materials and methods

### Animals

Male Sprague–Dawley (SD) adults (grade SPF, weighing 200 to 240 g) and pregnant SD rats (grade SPF) were obtained from Anhui Experimental Animal Center (Hefei, China). The rats were kept under standard lighting conditions (12-hour light/dark cycle). The procedure for animal surgery was performed in accordance with the Guidelines of Animal Care and Use Committee of Anhui Medical University.

### Materials

Specific mAb against MANF was prepared according to the method described previously [13]. Mouse anti-rat CD68 (catalogue number MCA341GA) was obtained from Serotec (Indianapolis, IN, USA). Mouse anti-NeuN (catalogue number MAB377) was obtained from Millipore (Billerica, MA, USA). Rabbit polyclonal to binding protein for immunoglobulins/glucose-regulated protein of 78 kDa (BIP/Grp78) antibody (catalogue number ab53068) was obtained from Abcam Ltd (Hongkong, China). Alexa Fluor-488 labeled anti-mouse IgG (catalogue number A11029) and Alexa Fluor-568 labeled anti-rabbit IgG (catalogue number A11036) were obtained from Invitrogen Corporation (Carlsbad, CA, USA). The 3,3^′^-diaminobenzidine tetrahydrochloride substrate was purchased from Vector Laboratories (Burlingame, CA, USA). The BCA Protein Assay Kit was from Thermo Fisher Scientific (Rockford, IL, USA). Horseradish peroxidase-conjugated anti-mouse and anti-rabbit IgG (catalogue number P0217) was from Dako (Glostrup, Denmark). MG132 was from Tocris Bioscience (Ellisville, MO, USA). All other antibodies and chemicals were obtained from Sigma-Aldrich (St Louis, MO, USA).

### Middle cerebral artery occlusion

The animal study was approved by the Animal Care and Use Committee in Anhui Medical University. All SD rats were treated according to the Guide for the Care and Use of Laboratory Animals. Male SD rats were obtained and bred as described previously [12]. The focal ischemia models were set up by middle cerebral artery occlusion as described previously [12]. Briefly, the rats were anesthetized and the right common carotid artery was exposed allowing the insertion of a nylon filament (0.235 mm in diameter) to the end of the internal carotid artery to block the origin of the right middle cerebral artery. Two hours or 4 hours after the occlusion, the nylon filament was withdrawn to allow reperfusion for 24 hours. The rats were sacrificed under deep anesthesia.

### Primary glial cell culture

Pregnant SD rats at embryonic days 16 to 18 were deeply anesthetized and the embryos were taken out. The cortexes and hippocampi were separated and placed in ice-cold Ca^2+^-free and Mg^2+^-free Hank’s solution. Cells were mechanically dissociated in a nutrient medium by triturating with a flame-polished sterile Pasteur pipette. Cell debris was removed by centrifugation. The cells were resuspended in DMEM containing 10% fetal bovine serum and 10% horse serum and plated onto 24-well plates precoated with poly-d-lysine. The cells were incubated in a humidified incubator at 37°C with 5% CO_2_ and the medium was changed every 2 or 3 days. After several days of culture, the cells were exposed to low serum (5%), MG132 (10 μmol/l), tunicamycin (1 μg/ml) for 24 hours. The cells were then collected for western blotting or fixed in phosphate-buffered 4% paraformaldehyde for immunofluorescent staining.

### Immunofluorescent staining

Adult SD rats were deeply anaesthetized with 10% chloral hydrate (3 ml/kg, intraperitoneally) and transcardially perfused with 4% paraformaldehyde in PBS (pH 7.4). Brains were then removed and subsequently placed in the same paraformaldehyde solution until further processing. The tissue was dehydrated through ethanol and xylene, and then embedded in paraffin. Four-micrometer coronal sections were processed for immunofluorescent staining using standard procedures. Briefly, brain sections were hydrated and rinsed in PBS. After antigen retrieval, sections were permeabilized/blocked in PBS containing 0.5% Triton X-100 and 5% goat serum. The sections were incubated with primary antibody overnight at 4°C. Negative controls were performed by substituting the primary antibody with PBS. MANF antibodies were prepared as described previously [12]. For dual fluorescent staining, the sections were incubated with Alexa Fluor 488-conjugated or 568-conjugated IgG (Invitrogen, Carlsbad, CA, USA) and observed under fluorescent microscopy (Olympus, Tokyo, Japan). Immunocytofluorescent staining was performed as described previously [12]. 4^′^,6-diamidino-2-phenylindole was used to stain the nuclei. The images were taken under a fluorescent microscope.

### Western blotting

Cultured cells were harvested and lysed with 10 volumes of 1×SDS sample buffer. The samples were boiled for 5 minutes and processed for SDS-PAGE and subsequent western blotting. Briefly, after blocking with 5% nonfat milk in PBS for 30 minutes, the membranes were incubated with primary and secondary antibodies for 1 hour at room temperature, respectively. The immunoreactive signals were visualized using the enhanced chemiluminescence kit from Pierce (Rockford, IL, USA). For the relatively quantitative analysis of western blot, the densitometry was carried out in Photoshop software (Adobe, San Jose, CA, USA). The levels of the proteins were normalized to the level of glyceraldehyde-3-phosphate dehydrogenase.

### Reverse transcription PCR

Total RNA was isolated with TRIZOL reagent (Invitrogen) according to the manufacturer’s instructions. Reverse transcription was performed with AMV from Promega (Madison, WI, USA) using the manufacturer’s protocol. Amplification primers were as follows: 5^′^-GGA GCT GGA AGC CTG GTA TGA-3^′^ (forward) and 5^′^-TCC CTG GTC AGG CGC TCG ATT T-3^′^ (reverse) for CCAAT/-enhancer-binding protein homologous protein (CHOP); 5^′^-GGT ATT GAA ACT GTG GGA GG-3^′^ (forward) and 5^′^-TTG TCT TCA GCT GTC ACT CG-3^′^ (reverse) for BIP; 5^′^-TCC GCT ACT GTA AGC AAG GT-3^′^ (forward) and 5^′^-CTT CAC CTA GGA TCT TGG TG-3^′^ (reverse) for MANF; and 5^′^-TCA AGA TCA TTG CTC CTC CTG AG-3^′^ (forward) and 5^′^-ACA TCT GCT GGA AGG TGG ACA-3^′^ (reverse) for β-actin.

### Propidium iodide staining

Propidium iodide staining was used to identify dead cells. Glial cells were grown on poly-d-lysine-coated coverslips and treated with ER stress inducers. The cells were then fixed in 70% ethanol for at least 30 minutes at 4°C. To ensure that only DNA was stained, cells were treated with 50 μl RNase (100 μg/ml). The cells were then stained with 200 μl propidium iodide (50 μg/ml). The images were taken under fluorescent microscopy.

### Statistical analysis

Data are expressed as the mean ± standard error of the mean. Multiple comparisons were statistically evaluated by a one-way analysis of variance between-groups test. A significance level of 5% was used in all statistical tests.

## Results

### Ischemia-induced MANF expression in the astrocytes

**Figure 1:**
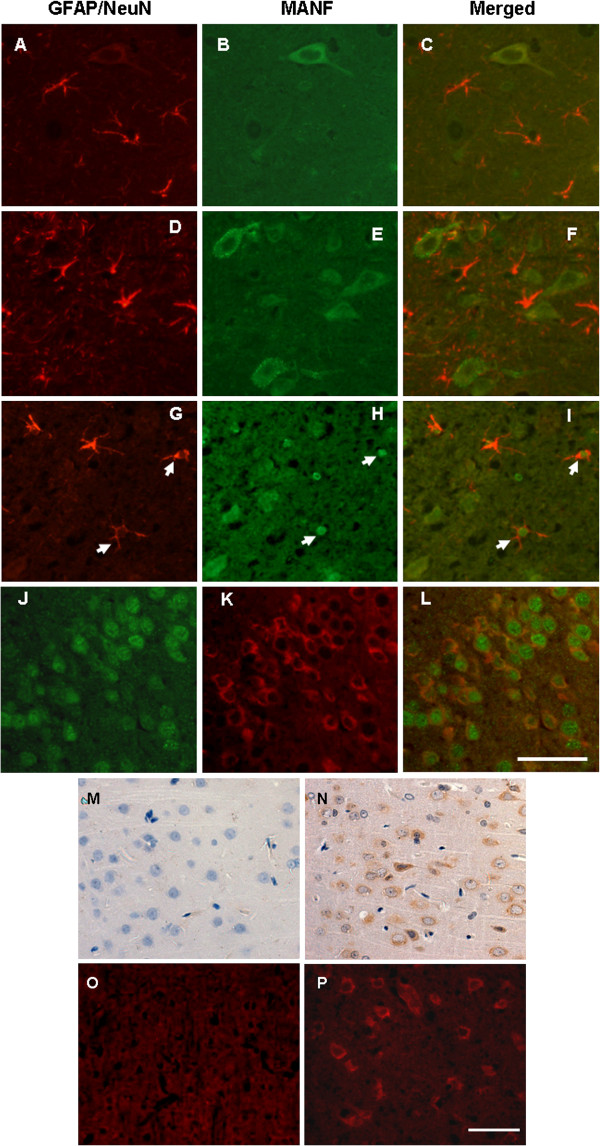
**Mesencephalic astrocyte-derived neurotrophic factor expression in the astrocytes of brain tissue.** Brain samples were collected from the normal (**A** to **C**) and ischemic rat brain tissue (**D** to **L**). (**D** to **F**) and (**J** to **L**) Two-hour ischemia followed by 24-hour reperfusion; (**G** to **I**) 4-hour ischemia followed by 24-hour reperfusion. (**A** to **I**) Mesencephalic astrocyte-derived neurotrophic factor (MANF) expression in astrocytes was detected by double immunofluorescent staining for glial fibrillary acidic protein (GFAP) (red, **A**, **D**, and **G**) and MANF (green, **B**, **E**, and **H**) with anti-GFAP and anti-MANF, respectively. (**J** to **L**) MANF expression in neurons was detected with anti-MANF (red, **K**). The neurons were identified by anti-NeuN (green, **J**). Ischemia-induced MANF expression was found predominantly in neurons (**K**). A small number of astrocytes expressing MANF are indicated by arrows. Scale bar = 50 μm. (**M** to **P**) Specificity of the antibody against MANF in brain tissues was detected by immunohistochemistry and immunofluorescent staining. Brain sections were incubated with primary antibody (anti-MANF) in (**N**) and (**P**). Negative controls were performed by substituting the primary antibody with PBS (**M** and **O**). Scale bar = 50 μm.

**Figure 2:**
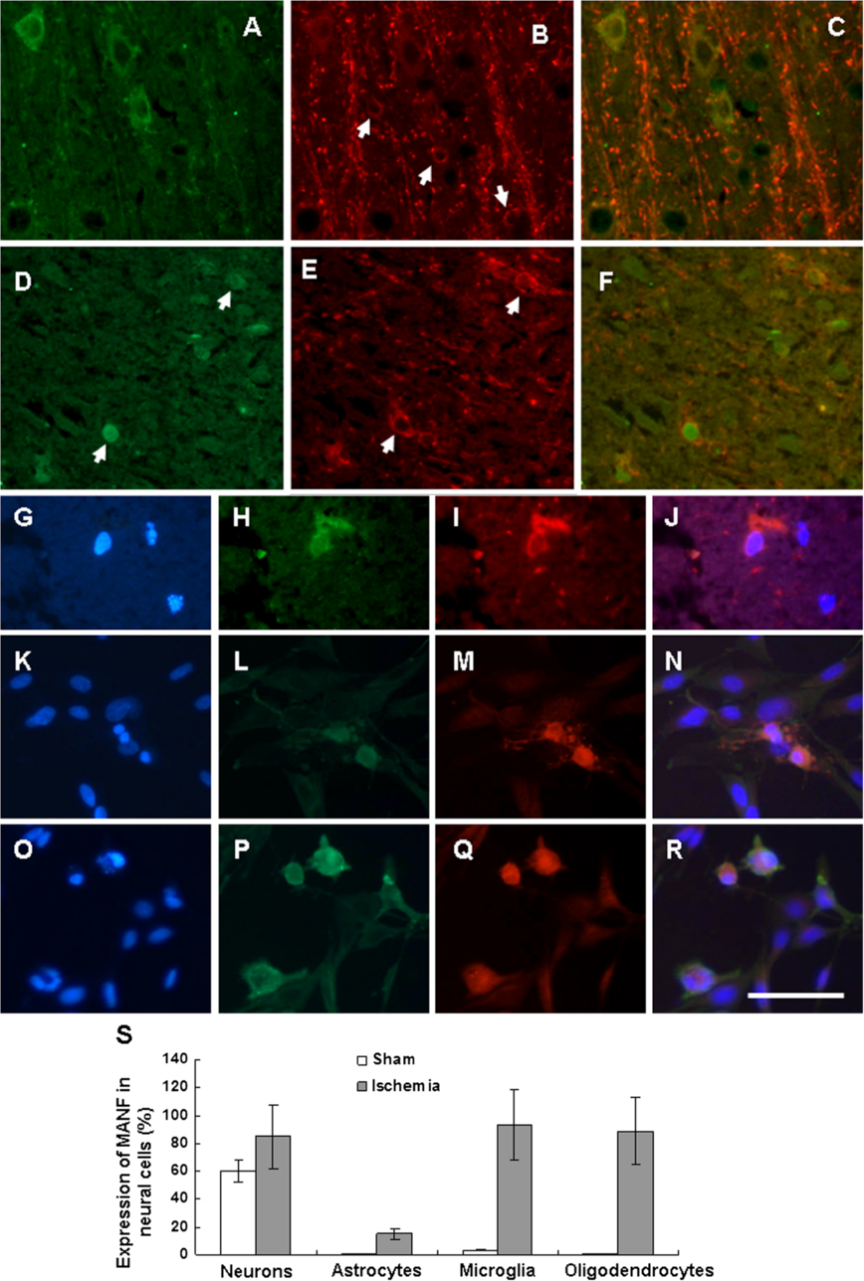
**Expression of mesencephalic astrocyte-derived neurotrophic factor in oligodendrocytes.** Mesencephalic astrocyte-derived neurotrophic factor (MANF) was expressed in normal cerebral cortex (**A** to **C**) and ischemic cerebral cortex (**D** to **J**). The primary cultured oligodendrocytes were treated with vehicle (DMEM medium containing 5% serum) (**K** to **N**) or tunicamycin (1 μg/ml) (**O** to **R**). Twenty-four hours after treatment, immunofluorescent staining was performed. The oligodendrocytes were identified with anti-2-3-cyclic nucleotide 3-phosphodiesterase (anti-CNP) (red, **B**, **E**, **I**, **M**, and **Q**). MANF was detected with anti-MANF antibody (green, **A**, **D**, **H**, **L**, and **P**). Scale bar = 50 μm. (**S**) Percentage of MANF-positive cells in neural cells. The number of MANF-positive cells and the number of double positive cells co-expressing glial fibrillary acidic protein (GFAP), CD68, CNP, or NeuN were counted in five randomly selected fields under a high-power field (×400 magnification). The percentage of MANF expressing cells in the population of astrocytes, microglial, and oligodendrocytes was calculated.

MANF was so named because it was originally isolated from a rat mesencephalic astrocyte line [1] . However, the pattern of MANF expression in astrocytes has not been reported. We first examined the expression of MANF in astrocytes by double-labeled immunofluorescent staining. Negative controls were performed to show the specificity of anti-MANF antibody in the brain tissue by substituting the primary antibody with PBS (Figure 1M,N,O,P). Glial fibrillary acidic protein and NeuN (a neuronal-specific nuclear protein) were used as the markers for astrocytes and neurons, respectively. To our surprise, unlike its name, MANF was predominately expressed in the NeuN-positive neurons (Figure 1J,K,L), but not in astrocytes (Figure 1A,B,C), in the normal rat cortex. Slight ischemia (focal ischemia for 2 hours) upregulated MANF expression in the glial fibrillary acidic protein-negative neural cells (Figure 1D,E,F). However, severe ischemia (focal ischemia for 4 hours with remarkable tissue edema) induced MANF expression in the astrocytes (Figure 1G,H,I, indicated by arrows). The percentage of MANF-positive cells in the astrocytes is shown in Figure 2S, and was less than that in microglia and oligodendrocytes.

**Figure 3:**
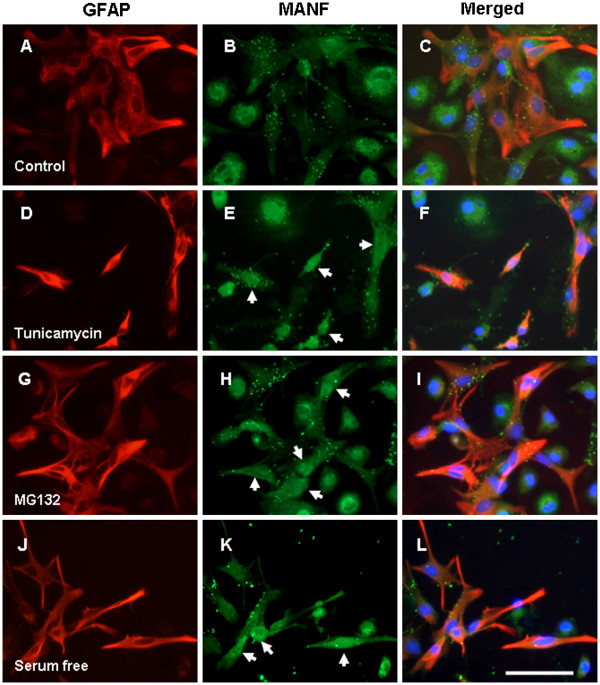
**Induction of mesencephalic astrocyte-derived neurotrophic factor expression in the primarily cultured astrocytes.** Cells cultured in the DMEM medium containing 5% serum were used as controls (**A** to **C**). Glial cells were cultured as described in Materials and methods and treated with 1 μg/ml tunicamycin (**D** to **F**), 10 μM MG132 (**G** to **I**), and serum-free DMEM medium (**J** to **L**), respectively. Twenty-four hours after treatment, the astrocytes were identified with anti-glial fibrillary acidic protein (anti-GFAP) antibody (red, **A**, **D**, **G**, and **J**). Mesencephalic astrocyte-derived neurotrophic factor (MANF) expression was detected with monoclonal anti-MANF antibody (green, **B**, **E**, **H**, and **K**). Nuclei were stained with 4^′^,6-diamidino-2-phenylindole (blue). The arrows show MANF immune-positive astrocytes after treatment. Scale bar = 50 μm.

**Figure 4:**
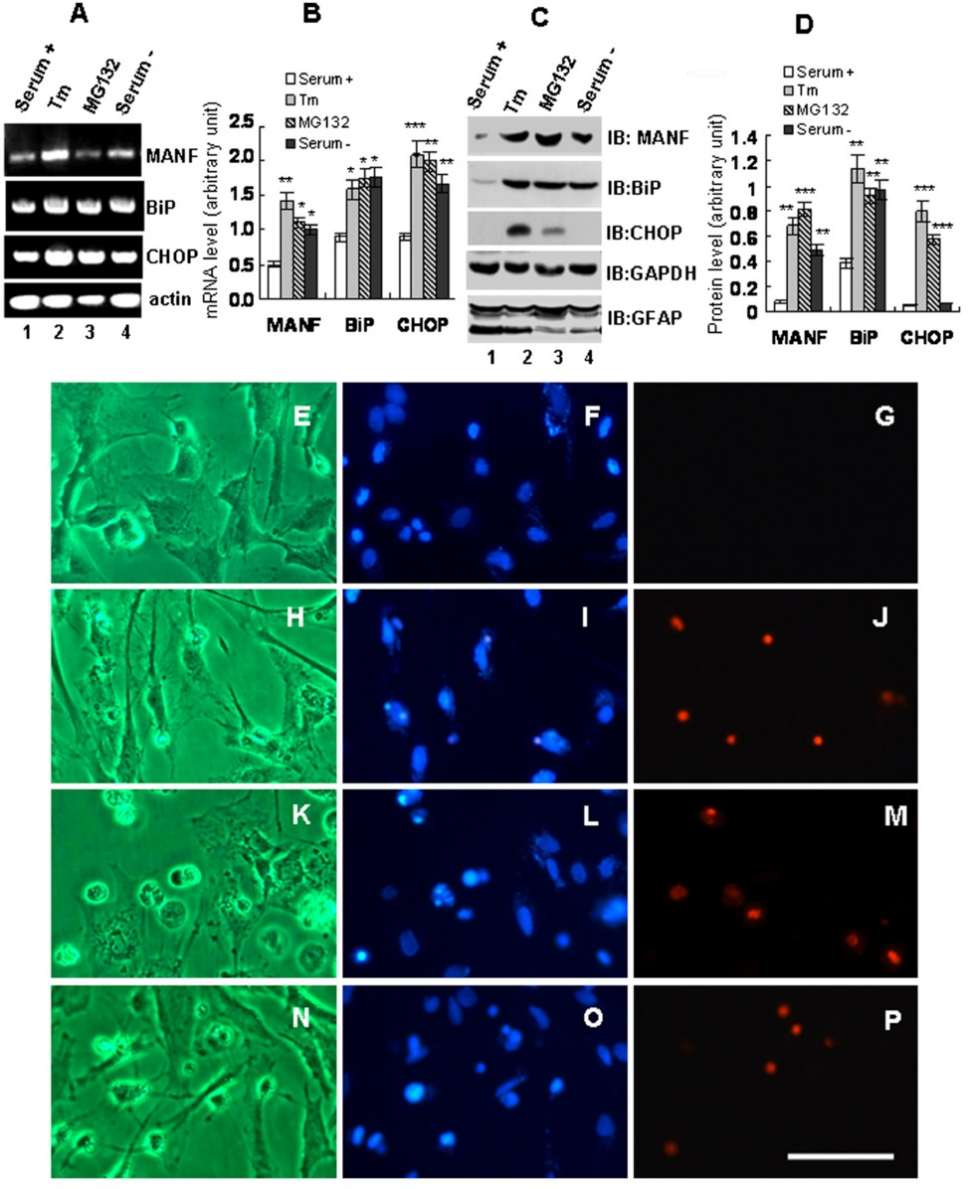
**Endoplasmic reticulum stress and mesencephalic astrocyte-derived neurotrophic factor expression in cultured primary glial cells. **(**A to D**) Glial cells in the mixed culture were treated as indicated (serum+ control, DMEM medium containing 5% serum; Tm, tunicamycin, 1 μg/ml; MG132, 10 μM; serum–, serum-free DMEM medium). Twenty-four hours after treatment, the cells were collected and processed for RT-PCR and immunoblotting (IB). Levels of mRNAs (**A**) and proteins (**C**) were quantitated and normalized by actin. The quantitative data in (**A**) and (**C**) are shown in (**B**) and (**D**), respectively. Values expressed as mean ± standard error of the mean of three independent experiments. *P <0.05, **P <0.01, and ***P <0.001, compared with serum+ control. (**E** to **P**) Glial cells were treated with serum+ (**E** to **G**), Tm (**H** to **J**), MG132 (**K** to **M**), and serum– (**N** to **P**). Twenty-four hours after treatment, the cells were stained with 4^′^,6-diamidino-2-phenylindole (**F**, **I**, **L**, and **O**) and propidium iodide (**G**, **J**, **M**, and **P**), and then observed with a microscope under bright field (**E**, **H**, **K**, and **N**) and fluorescence. Scale bar = 50 μm.

Further study was carried out in cultured primary astrocytes. Consistently, MANF was almost undetectable in astrocytes cultured in medium containing 5% fetal bovine serum (Figure 3A,B,C), but it was induced in cells treated with ER stress inducers, including tunicamycin (an inhibitor of protein glycosylation) (Figure 3D,E,F, indicated by arrows), proteasome inhibitor MG132 (Figure 3G,H,I, indicated by arrows), and nutrition starvation with serum-free culture medium (Figure 3J,K,L, indicated by arrows). The upregulation was further supported by the increases in MANF mRNA and protein as revealed by RT-PCR and western blotting, respectively (Figure 4A,B,C,D). These results suggest that expression MANF is inducible under stress condition in astrocytes but is constitutively expressed in neurons.

### Ischemia-induced microglial activation and MANF expression

**Figure 5:**
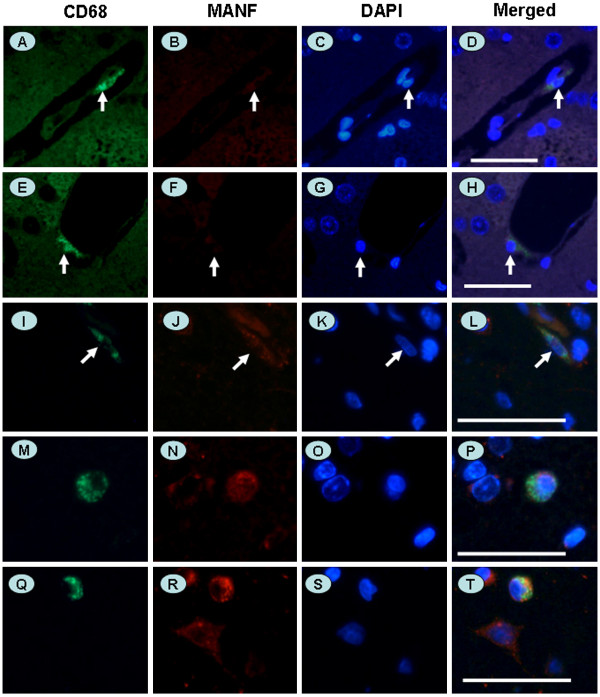
**Differential expression of mesencephalic astrocyte-derived neurotrophic factor in the microglia in brain tissue.** (**A** to **D**) A rod-shaped microglial cell within blood vessel in the normal cerebral cortex. (**E** to **H**) A ramified microglial cell under vascular endothelium in the normal cerebral cortex. (**I** to **L**) A rod-shaped microglial cell with weak mesencephalic astrocyte-derived neurotrophic factor (MANF)-positive immunoreaction in the ischemic cerebral cortex. (**M** to **P**) An amoeboid microglial cell with MANF-positive immunoreaction in the ischemic cerebral cortex. (**Q** to **T**) A round microglial cell with strong MANF-positive immunoreaction in the ischemic cerebral cortex. Microglial cells were identified with anti-CD68 antibody (green, **A**, **E**, **I**, **M**, and **Q**). MANF was detected with monoclonal anti-MANF (red, **B**, **F**, **J**, **N**, and **R**). Nuclei were stained by 4^′^,6-diamidino-2-phenylindole (blue, **C**, **G**, **K**, **O**, and **S**). Scale bar = 50 μm.

**Figure 6:**
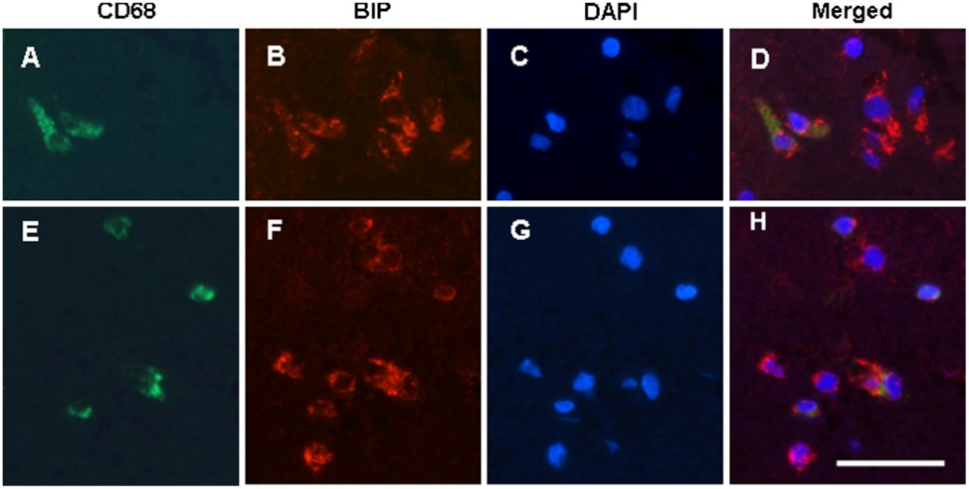
**BIP induction in the microglia in the ischemic cerebral cortex.** (**A** to **D**) Ramified microglial cells in the ischemic cerebral cortex. (**E** to **H**) Round microglial cells in the ischemic cerebral cortex. Microglial cells were identified with anti-CD68 antibody (green, **A** and **E**). BIP was detected with rabbit anti-BIP (red, **B** and **F**). Nuclei were stained by 4^′^,6-diamidino-2-phenylindole (blue, **C** and **G**). Scale bar = 50 μm.

Activation of microglia has been found in acute and chronic neuroinflammation and neurodegenerative diseases. To learn more about MANF expression in glial cells, we also detected MANF in microglia using immunofluorescent double staining with anti-MANF and anti-CD68, a marker of microglia. There were few CD68-positive microglia cells in the cortex, except for those that appeared in the small vessels (Figure 5A to H, indicated by arrows), and these cells did not express detectable levels of MANF. However, many CD68-positive microglia were found in the ischemic cortex with detectable MANF (Figure 5I to T). Additionally, the expression of MANF in microglia depended on the morphologies of microglia. For example, MANF-positive microglial cells were slightly rod-shaped or ramified (Figure 5I,J,K,L, indicated by arrows). However, the amoeboid-like or round microglial cells displayed a strong MANF immunostaining (Figure 5M to T). The induction of BIP/Grp78 was also observed in both the rod-shaped and round microglial cells in the ipsilateral ischemic cortex, but not in the contralateral nonischemic cortex (Figure 6A to H), suggesting ER stress is involved in ischemia-induced microglial activation.

**Figure 7:**
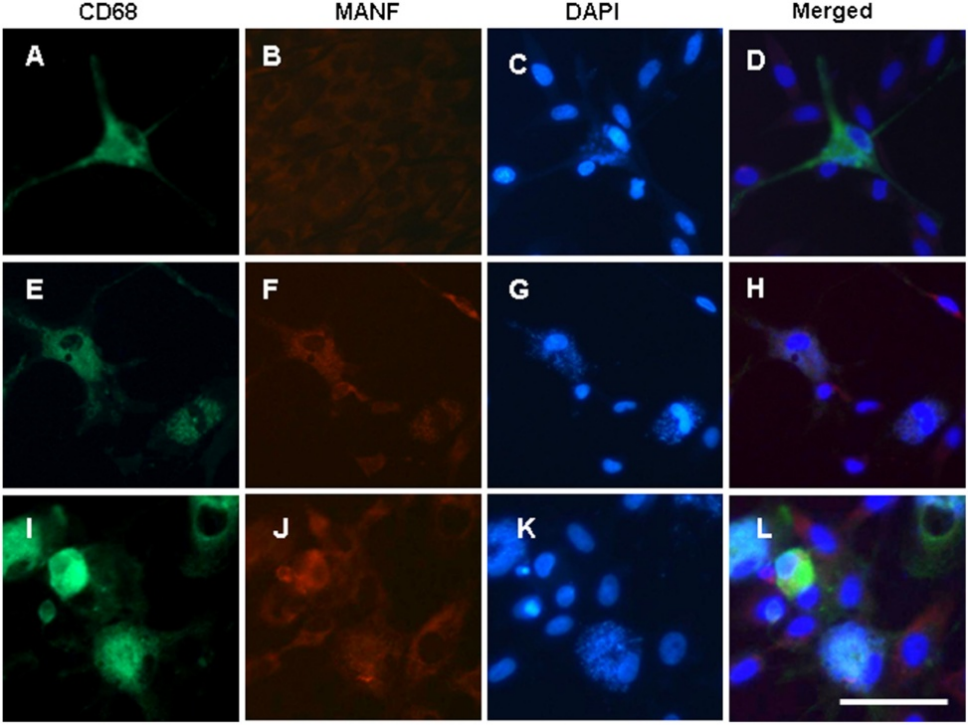
**Mesencephalic astrocyte-derived neurotrophic factor expression in cultured primary microglia.** Glial cells were cultured as described in Materials and methods. The serum concentration in DMEM was lowered from 10% to 5% during treatment. Immunofluorescent staining was performed in vehicle controls (**A** to **D**) and 24 hours after treatment with 1 μg/ml tunicamycin (**E** to **L**). Microglia were identified with anti-CD68 antibody (green, **A**, **E**, and **I**). Mesencephalic astrocyte-derived neurotrophic factor (MANF) expression was detected with monoclonal anti-MANF antibody (red, **B**, **F**, and **J**). Nuclei were stained by 4^′^,6-diamidino-2-phenylindole (blue, **C**, **G**, and **K**). Scale bar = 50 μm.

Similar patterns of MANF expression were observed in cultured primary microglia, but not in the ramified microglial cells (Figure 7A,B,C,D). After exposure to tunicamycin for 24 hours, the shapes of microglia were changed to amoeboid (Figure 7E) or round (Figure 7I), and MANF expression was upregulated (Figure 7F,J). These results support the findings described *in vivo*.

### Ischemia-induced MANF expression in oligodendrocytes

The expression of MANF was also determined in oligodendrocytes in the present study. The myelin protein 2^′^3^′^-cyclic nucleotide 3^′^-phosphodiesterase (CNP) was used as a marker of oligodendrocytes. In the normal cortex, a large amount of branched processes and a small amount of cell bodies were detected, and the CNP-positive fibers were thicker and arranged in an ordered radial pattern (Figure 2B, arrows show the soma). MANF, however, was expressed in the soma (Figure 2D, indicated by arrows), and the amount of branched processes was reduced in the ischemic cortex (Figure 2E). When double-labeled with 4^′^,6-diamidino-2-phenylindole, a small amount of MANF was found in the nuclei (Figure 2G,H,I,J). As in the astrocytes and microglia, MANF expression was upregulated in oligodendrocytes after treatment with tunicamycin (Figure 2O,P,Q,R). These results indicate that MANF was induced in oligodendrocytes not only by focal cerebral ischemia but also by ER stress.

### Induction of ER stress and MANF expression in a mixed culture of the primary glial cells

Our previous study demonstrated that MANF is an ER stress inducible protein [8]. Here we have shown that MANF was induced in the glial cells that were exposed to a variety of stimuli. BIP and CHOP were used as the markers of ER stress. As we predicted, the levels of BIP and CHOP were significantly upregulated both in mRNA transcription (Figure 4A,B) and in protein translation (Figure 4C,D) after the cells were treated with tunicamycin and MG132. However, nutrient starvation (serum removal) exerted a lesser effect on the protein level of CHOP. Similarly, the expression of MANF mRNA and protein was increased in response to the treatment. These results suggest that induction of ER stress upregulates MANF in glial cells.

CHOP, also known as growth arrest-inducible and DNA damage-inducible gene 153 (GADD153), is the proapoptotic protein that mediates ER stress-induced apoptosis [14]. CHOP has been reported to play a pivotal role in astrocyte death induced by oxygen and glucose deprivation [15]. We were wondering whether the ER stress inducers could cause glial cell death. To test this, the dead cells were detected by propidium iodide staining and 4^′^,6-diamidino-2-phenylindole staining. The morphology of the glial cell was also observed under microscope. The number of dead cells increased after treatment with tunicamycin (Figure 4J) or MG132 (Figure 4M), or under serum-free culture condition (Figure 4P), suggesting that ER stress induces glial cell death.

## Discussion

We report in this article the expression patterns of MANF in glial cells *in vivo* and *in vitro*. For the first time the characteristics of MANF expression in the different types of glial cells were revealed, and they were not as expected given the history of MANF. Unlike its name ’mesencephalic astrocyte-derived neurotrophic factor’ suggests, the astrocytes were not the major source of MANF in the brain tissue. Although there was a small amount of MANF expression in normal tissue, the MANF-positive cells were neurons, not astrocytes. However, severe cerebral ischemia could induce MANF expression in glial cells, including astrocytes and oligodendrocytes. MANF was significantly upregulated in neurons even by slight cerebral ischemia [12], suggesting that the neurons are a major source of MANF in the brain tissue. Nevertheless, MANF expression was easily induced by ER stress inducers and nutrition deprivation in the cultured primary glial cells, including astrocytes, microglia, and oligodendrocytes, suggesting that the expression of MANF in glial cells is stress inducible. Collectively, these results indicate that MANF can be induced and differentially expressed in glial cells, and that neurons are the major source of MANF in the brain tissue.

Glial cells are the major population of cells in the brain. All these glial cells synergistically supply nutrition, maintain homeostasis, and participate in signal transmission in the central nervous system (CNS). Astrocytes, the major glial cell type in the CNS, are associated with both neuroprotection and cytotoxicity when they are activated in response to toxic substances or disease states. Astrocyte activation is one of the key components of cellular responses to brain injuries and neurodegeneration [16]. We found that severe cerebral ischemia in rats and ER stress inducers *in vitro* induced MANF expression in astrocytes. The pattern of MANF expression in astrocytes was different from that in other glial cells and neurons, although it is still unclear why. This difference in expression might be associated with the function of astrocytes. Additionally, we also observed glial cell death induced by ER stress *in vitro*. Nevertheless, further investigation will be needed to determine whether the expression of MANF in the astrocytes is neuroprotective or neurodegenerative.

Microglia, the resident macrophages of the brain, are usually kept in a quiescent ramified state under physiological conditions. The microglia are extremely plastic, and undergo a variety of morphological changes according to their location and current role. A variety of stimuli, including substances released by damaged neurons, invading pathogens, phagocytosing debris, and released proinflammatory mediators, can induce the morphological changes of microglia. Each form of microglia is thought to play a distinct functional role [17,18]. In this study, we found that ischemia-induced MANF expression in microglia depends on the state of microglia. MANF was only expressed in the activated microglia in the tissue, such as the amoeboid-shaped or round-shaped microglia. However, ischemia-induced microglial aggregation in the cerebral cortex and hippocampal dentate gyrus did not upregulate MANF expression (data not shown) despite the fact that microglial aggregation in the hippocampal dentate gyrus is a marker of mild hypoxic-ischemic brain insult [19]. The relationship between MANF induction and microglia activation is not yet clear. Furthermore, the induction of BIP/Grp78 was observed in both rod-shaped and round microglial cells in the ipsilateral ischemic cortex, but not in the contralateral nonischemic cortex, suggesting ER stress is involved in ischemia-induced microglial activation.

The cascade of microglial activation is a fine-tuned process that is also regulated by factors derived from neurons and other glial populations, particularly astrocytes. For example, astrocytes can induce the transformation of amoeboid microglia into ramified microglial cells and reduce proliferative activity [20]. The presence of activated microglia is linked to increased neuronal damage. In contrast, ablation of microglia is also associated with increased damage [21], which suggests that microglia play a complex part in the etiology of neuronal injury. CD68 (also called ED-1) and Iba-1 were usually used to identify the microglial cells. Iba-1 can recognize resting as well as activated microglia. CD68 was also used as a marker of microglia [22-25]. High levels of CD68 expression are associated with activated microglia, whilst low levels of expression are associated with quiescent ramified microglia [22-24,26]. CD68-positive cells were present in all four types of morphology [27], which was consistent with our findings described in this study.

Oligodendrocytes are essential for the proper development and function of axonal networks in the CNS. During development, these myelin-forming cells are metabolically the most active cells in the CNS [28]. The main proteins of myelin, such as myelin basic protein and CNP, interact with microtubules and microfilaments in oligodendrocytes [29]. Our study found that ischemia and ER stress induced MANF expression in the oligodendrocytes, accompanied by a decrease in processes. However, the exact role of MANF needs further investigation. Popko’s group reported that severe ER stress induced by IFNγ in myelinating oligodendrocytes during development caused oligodendrocyte apoptosis [30]. Nevertheless, modest ER stress induced by IFNγ in mature oligodendrocytes of adult mice protected against experimental autoimmune encephalomyelitis-induced demyelination, axonal damage, and oligodendrocyte loss [31].

In recent years, several neurotrophic factors such as brain-derived neurotrophic factor and glial cell line-derived neurotrophic factor have been found and known to regulate synaptic plasticity in the CNS [32,33]. MANF is a novel neurotrophic factor and forms a novel evolutionally conserved protein family along with CDNF [7]. Intracortical delivery of recombinant MANF protein [34] or encoding MANF adeno-associated virus [35] protected tissue from ischemic brain injury *in vivo*. Our previous study had shown that recombinant human MANF was protective to neurons [12]. These results suggest that induction of MANF is probably protective to neural cells. Recently, the crystal structure of MANF has revealed a well-defined N-terminal domain belonging to the saposin family and a mostly disordered C-terminal domain, which support the bi-functional role of MANF. The C-terminal domain of MANF is homologous to the SAP domain of Ku70, a well-known inhibitor of pro-apoptotic Bcl-2-associated X protein (Bax) [9]. Cellular studies have demonstrated that MANF protected neurons intracellularly as efficiently as Ku70 [9].

In this study we also found that both ER stress inducer and nutrition deprivation upregulated BIP and CHOP and caused glial death, which was similar to the findings described by Oyadomari’s and Benavides’ groups [14,15]. CHOP is the first protein identified that mediates ER stress-induced apoptosis and much is known on the roles of this molecule in apoptosis. CHOP could also be induced by nutrient depletion such as glucose deprivation and amino acid starvation [14]. CHOP favors a pro-apoptotic drive at the mitochondria by proteins that cause mitochondrial damage, cytochrome C release, and caspase-3 activation. The target genes for CHOP include growth and DNA damage protein 34 and ER oxidoreductin 1, which promote recovery from ER stress-mediated translational repression in the ER.

## Conclusions

This study demonstrated the patterns and characteristics of MANF expression in different types of glial cells. The results suggest that upregulated MANF expression is associated with activated glial cells, which will help us to understand the function of MANF and the mechanisms of ischemia-induced neural injury.

## Abbreviations

BIP/Grp78: Binding protein for immunoglobulins/glucose-regulated protein of 78 kDa; CDNF: Conserved dopamine neurotrophic factor; CHOP: CCAAT/-enhancer-binding protein homologous protein; CNP: 2^′^3^′^-cyclic nucleotide 3^′^-phosphodiesterase; CNS: Central nervous system; DMEM: Dulbecco’s modified eagle’s medium; ER: Endoplasmic reticulum; IFN: Interferon; mAb: Monoclonal antibody; MANF: Mesencephalic astrocyte-derived neurotrophic factor; PBS: Phosphate-buffered saline; PCR: Polymerase chain reaction; SD: Sprague–Dawley.

## Competing interests

The authors declare that they have no competing interests.

## Authors’ contributions

YXS designed the research and wrote the paper. YJS, AMS, YHW, DQC, HPW, FCW, LJF, SYF performed the research. All authors read and approved the final manuscript.

## References

[B1] Petrova P Raibekas A Pevsner J Vigo N Anafi M Moore MK Peaire AE Shridhar V Smith DI Kelly J Durocher Y Commissiong JW MANF: a new mesencephalic, astrocyte-derived neurotrophic factor with selectivity for dopaminergic neurons J Mol Neurosci 2003 20 173 188 10.1385/JMN:20:2:173 12794311

[B2] Shridhar V Rivard S Shridhar R Mullins C Bostick L Sakr W Grignon D Miller OJ Smith DI A gene from human chromosomal band 3p21.1 encodes a highly conserved arginine-rich protein and is mutated in renal cell carcinomas Oncogene 1996 12 1931 1939 8649854

[B3] Shridhar R Shridhar V Rivard S Siegfried JM Pietraszkiewicz H Ensley J Pauley R Grignon D Sakr W Miller OJ Smith DI Mutations in the arginine-rich protein gene, in lung, breast, and prostate cancers, and in squamous cell carcinoma of the head and neck Cancer Res 1996 56 5576 5578 8971156

[B4] Evron E Cairns P Halachmi N Ahrendt SA Reed AL Sidransky D Normal polymorphism in the incomplete trinucleotide repeat of the arginine-rich protein gene Cancer Res 1997 57 2888 2889 9230196

[B5] Mizobuchi N Hoseki J Kubota H Toyokuni S Nozaki J Naitoh M Koizumi A Nagata K ARMET is a soluble ER protein induced by the unfolded protein response via ERSE-II element Cell Struct Funct 2007 32 41 50 10.1247/csf.07001 17507765

[B6] Parkash V Lindholm P Peränen J Kalkkinen N Oksanen E Saarma M Leppänen VM Goldman A The structure of the conserved neurotrophic factors MANF and CDNF explains why they are bifunctional Protein Eng Des Sel 2009 22 233 241 10.1093/protein/gzn080 19258449

[B7] Hoseki J Sasakawa H Yamaguchi Y Maeda M Kubota H Kato K Nagata K Solution structure and dynamics of mouse ARMET FEBS Lett 2010 584 1536 1542 10.1016/j.febslet.2010.03.008 20214902

[B8] Lindholm P Saarma M Novel CDNF/MANF family of neurotrophic factors Dev Neurobiol 2010 70 360 371 20186704 10.1002/dneu.20760

[B9] Hellman M Arumae U Yu LY Lindholm P Peranen J Saarma M Permi P Mesencephalic astrocyte-derived neurotrophic factor (MANF) has a unique mechanism to rescue apoptotic neurons J Biol Chem 2011 286 2675 2680 10.1074/jbc.M110.146738 21047780 PMC3024763

[B10] Apostolou A Shen Y Liang Y Luo J Fang S Armet, a UPR-upregulated protein, inhibits cell proliferation and ER stress-induced cell death Exp Cell Res 2008 314 2454 2467 10.1016/j.yexcr.2008.05.001 18561914 PMC6719340

[B11] Lindholm P Peranen J Andressoo JO Kalkkinen N Kokaia Z Lindvall O Timmusk T Saarma M MANF is widely expressed in mammalian tissues and differently regulated after ischemic and epileptic insults in rodent brain Mol Cell Neurosci 2008 39 356 371 10.1016/j.mcn.2008.07.016 18718866

[B12] Yu YQ Liu LC Wang FC Liang Y Cha DQ Zhang JJ Shen YJ Wang HP Fang S Shen YX Induction profile of MANF/ARMET by cerebral ischemia and its implication for neuron protection J Cereb Blood Flow Metab 2010 30 79 91 10.1038/jcbfm.2009.181 19773801 PMC2949098

[B13] Wang FC Wang HP Li Q Fang S Shen YX Prokaryotic expression of human ARMET and preparation of monoclonal antibodies against recombinant Harmet Acta Universitatis Medicinalis Anhui 2009 44 665 669

[B14] Oyadomari S Mori M Roles of CHOP/GADD153 in endoplasmic reticulum stress Cell Death Differ 2004 11 381 389 10.1038/sj.cdd.4401373 14685163

[B15] Benavides A Pastor D Santos P Tranque P Calvo S CHOP plays a pivotal role in the astrocyte death induced by oxygen and glucose deprivation Glia 2005 52 261 275 10.1002/glia.20242 16001425

[B16] Hall ED Oostveen JA Gurney ME Relationship of microglial and astrocytic activation to disease onset and progression in a transgenic model of familial ALS Glia 1998 23 249 256 10.1002/(SICI)1098-1136(199807)23:3<249::AID-GLIA7>3.0.CO;2-# 9633809

[B17] Davis EJ Foster TD Thomas WE Cellular forms and functions of brain microglia Brain Res Bull 1994 34 73 78 10.1016/0361-9230(94)90189-9 8193937

[B18] Raivich G Bohatschek M Kloss CU Werner A Jones LL Kreutzberg GW Neuroglial activation repertoire in the injured brain: graded response, molecular mechanisms and cues to physiological function Brain Res Brain Res Rev 1999 30 77 105 10.1016/S0165-0173(99)00007-7 10407127

[B19] Del Bigio MR Becker LE Microglial aggregation in the dentate gyrus: a marker of mild hypoxic-ischaemic brain insult in human infants Neuropathol Appl Neurobiol 1994 20 144 151 10.1111/j.1365-2990.1994.tb01173.x 8072645

[B20] Jones LL Kreutzberg GW Raivich G Transforming growth factor beta’s 1, 2 and 3 inhibit proliferation of ramified microglia on an astrocyte monolayer Brain Res 1998 795 301 306 10.1016/S0006-8993(98)00325-4 9622658

[B21] Lalancette-Hebert M Gowing G Simard A Weng YC Kriz J Selective ablation of proliferating microglial cells exacerbates ischemic injury in the brain J Neurosci 2007 27 2596 2605 10.1523/JNEUROSCI.5360-06.2007 17344397 PMC6672496

[B22] Graeber MB Banati RB Streit WJ Kreutzberg GW Immunophenotypic characterization of rat brain macrophages in culture Neurosci Lett 1989 103 241 246 10.1016/0304-3940(89)90106-7 2682390

[B23] Graeber MB Streit WJ Kreutzberg GW The third glial cell type, the microglia: cellular markers of activation in situ Acta Histochem Suppl 1990 38 157 160 2080239

[B24] Slepko N Levi G Progressive activation of adult microglial cells in vitro Glia 1996 16 241 246 10.1002/(SICI)1098-1136(199603)16:3<241::AID-GLIA6>3.0.CO;2-4 8833194

[B25] Wang D Hazell AS Microglial activation is a major contributor to neurologic dysfunction in thiamine deficiency Biochem Biophys Res Commun 2010 402 123 128 10.1016/j.bbrc.2010.09.128 20932820

[B26] Kingham PJ Cuzner ML Pocock JM Apoptotic pathways mobilized in microglia and neurones as a consequence of chromogranin A-induced microglial activation J Neurochem 1999 73 538 547 10428049 10.1046/j.1471-4159.1999.0730538.x

[B27] Sanchez-Guajardo V Febbraro F Kirik D Romero-Ramos M Microglia acquire distinct activation profiles depending on the degree of alpha-synuclein neuropathology in a rAAV based model of Parkinson’s disease PLoS One 2010 5 e8784 10.1371/journal.pone.0008784 20098715 PMC2808388

[B28] Jiang S Seng S Avraham HK Fu Y Avraham S Process elongation of oligodendrocytes is promoted by the Kelch-related protein MRP2/KLHL1 J Biol Chem 2007 282 12319 12329 17324934 10.1074/jbc.M701019200

[B29] Richter-Landsberg C The cytoskeleton in oligodendrocytes. Microtubule dynamics in health and disease J Mol Neurosci 2008 35 55 63 10.1007/s12031-007-9017-7 18058074

[B30] Lin W Harding HP Ron D Popko B Endoplasmic reticulum stress modulates the response of myelinating oligodendrocytes to the immune cytokine interferon-gamma J Cell Biol 2005 169 603 612 10.1083/jcb.200502086 15911877 PMC2171696

[B31] Lin W Bailey SL Ho H Harding HP Ron D Miller SD Popko B The integrated stress response prevents demyelination by protecting oligodendrocytes against immune-mediated damage J Clin Invest 2007 117 448 456 10.1172/JCI29571 17273557 PMC1783809

[B32] Lu B Pang PT Woo NH The yin and yang of neurotrophin action Nat Rev Neurosci 2005 6 603 614 10.1038/nrn1726 16062169

[B33] Lin LF Doherty DH Lile JD Bektesh S Collins F GDNF: a glial cell line-derived neurotrophic factor for midbrain dopaminergic neurons Science 1993 260 1130 1132 10.1126/science.8493557 8493557

[B34] Airavaara M Shen H Kuo CC Peranen J Saarma M Hoffer B Wang Y Mesencephalic astrocyte-derived neurotrophic factor reduces ischemic brain injury and promotes behavioral recovery in rats J Comp Neurol 2009 515 116 124 10.1002/cne.22039 19399876 PMC2723810

[B35] Airavaara M Chiocco MJ Howard DB Zuchowski KL Peranen J Liu C Fang S Hoffer BJ Wang Y Harvey BK Widespread cortical expression of MANF by AAV serotype 7: localization and protection against ischemic brain injury Exp Neurol 2010 225 104 113 10.1016/j.expneurol.2010.05.020 20685313 PMC2925275

